# An Efficient AP-ANN-Based Multimethod Fusion Model to Detect Stress through EEG Signal Analysis

**DOI:** 10.1155/2022/7672297

**Published:** 2022-12-12

**Authors:** Saranya K, Jayanthy S

**Affiliations:** ^1^Department of Information Science and Engineering, Kumaraguru College of Technology, Coimbatore 641049, India; ^2^Department of Electronics and Communication Engineering, Sri Ramakrishna Engineering College, Coimbatore 641022, India

## Abstract

Stress is a universal emotion that every human experiences daily. Psychologists say stress may lead to heart attack, depression, hypertension, strokes, or even sudden death. Many technical explorations like stress detection through facial expression, speech, text, physical behaviors, etc., were explored, but no consensus has been reached on the best method. The advancement in biomedical engineering yielded a rapid development of electroencephalogram (EEG) signal analysis that has inspired the idea of a multimethod fusion approach for the first time which employs multiple techniques such as discrete wavelet transform (DWT) for de-noising, adaptive synthetic sampling (ADASYN) for class balancing, and affinity propagation (AP) as a stratified sampling model along with the artificial neural network (ANN) as the classifier model for human emotion classification. From the EEG recordings of the DEAP dataset, the artifacts are removed, the signal is decomposed using a DWT, and features are extracted and fused to form the feature vector. As the dataset is high-dimensional, feature selection is done and ADASYN is used to address the imbalance of classes resulting in large-scale data. The innovative idea of the proposed system is to perform sampling using affinity propagation as a stratified sampling-based clustering algorithm as it determines the number of representative samples automatically which makes it superior to the K-Means, K-Medoid, that requires the K-value. Those samples are used as inputs to various classification models, the comparison of the AP-ANN, AP-SVM, and AP-RF is done, and their most important five performance metrics such as accuracy, precision, recall, F1-score, and specificity were compared. From our experiment, the AP-ANN model provides better accuracy of 86.8% and greater precision of 85.7%, a higher F1 score of 84.9%, a recall rate of 84.1%, and a specificity value of 89.2% which altogether provides better results than the other existing algorithms.

## 1. Introduction

Stress is a major problem experienced by humans in their daily life. Stress is defined as the way by which the body responds to a situation or threats. In the present situation with COVID-19 completely ruling the world, chronic stress has become very common among people, as the survey tells more than 70% of Americans experience stress regularly [1]. The most dangerous truth about stress is that people easily attain it being unaware of its effect on them. There are lots of causes for a person to be under stress in the current decade. The condition along with the situation that causes stress is generally named stressors that have a major influence on mood, health, and behavior [2]. The stressors can be work stress which is caused by a heavy workload, more responsibility, and risk of termination, or life stress, which is caused by unemployment, death of a person, and illness.

Stress cannot only be caused by external factors but also by internal such as constantly worrying about things that happen around. Stress is individual-specific as the amount of stress a person can tolerate varies due to many reasons [3]. Chronic stress occurs when a person experiences continuous stress without any relief. The stress which hurts humans is referred to as negative stress [4], and it may lead to various physical imbalances which include headache, high blood pressure, stroke, and heart attack and emotional imbalances which include depression, anxiety, hypertension, and fear. Sometimes, the stress may even lead to death. Hence, there is a primary need to detect the stress in the early stage and manage it through appropriate measures.

There are various methods in existence to analyze stress which includes analysis of stress in voice [5], detection of stress using image processing which is a system that detects stress by analyzing facial expression [6], and analysis of human stress by investigating mobile phones that is a design of collecting of information or data from smartphones, surveys, and call logs [7]. The traditional methods using the EEG signals have numerous drawbacks which include external factors such as sweating, room temperature, and invasive procedure. Therefore, there is a need for a method that is precise, accurate, noninvasive, and reliable. The proposed work aims in creating an EEG-based stress analyst, a system used to detect human stress levels using a noninvasive brain-computer interface.

There are numerous techniques proposed to detect stress from negative emotions like sadness and anger that were detected by classifying the EEG signals, and this method of using the EEG signals to detect emotions gives a promising result [8, 9]. Among all the noninvasive techniques [10] to determine brain activities, an EEG-based methodology was found to be best with a low setup cost. It measures the brain's electrical activity directly from electrodes that were laid on the scalp of the brain [11]. EEG measures the minute electrical difference produced by neurons using the electrodes and sends signals to the external device. With the enhancement in technology, massive development is attained in wearable systems that can record electrophysiological signals to detect acute stress [12]. The EEG signals are categorized into the number of sub-bands of different frequencies, and different brain states can be analyzed from each frequency band [13]. Stress is detected by classifying emotions using the machine learning algorithms from the recorded EEG signals [14].

In the proposed model, the DEAP dataset [15] which is the recording of EEG and peripheral physiological signals of 32 participants when they watch 40 one-minute-long videos is used. As not, all the channels contributed to the recognition of emotion, the existing channel selection methods have been analyzed [16, 17], and the ES method is being chosen for the channel selection in the proposed work. The effective technique used to recognize the original brain signals from various artifacts is the DWT, and it is one of the most widely used methods to decompose the original EEG signal into frequency bands that are functionally distinct such as delta (0.5–4 Hz), theta (4–8 Hz), alpha (8–12 Hz), beta (12–30 Hz), and gamma (30–100 Hz). The DWT provides more efficiency than other conventional methods in the separation of waves [18]. The DWT filtering is best because it considers both frequency (spectrum) and time (rhythmicity) features whereas the other de-noising methods consider only frequency [19, 20].

Since the dataset is of high dimensional with *n* number of features, an efficient feature selection method is needed to minimise the features that do not contribute much to the classified result. This is done with the help of the Pearson correlation coefficient (PCC) method in which the correlation between a feature-to-feature and feature-to-class are calculated. Then, the correlations between features are ranked in decreasing order. Then, the first feature is selected, and the feature set is expanded by adding the next feature in the order [21]. Then, the process is continued until there is no improvement and the best feature set is received. From the feature set, a range value is calculated, the features not in the range are eliminated, and the top 10 features alone are being used for the further process [22, 23].

The ADASYN algorithm is used in the proposed model to handle the class balance of the DEAP dataset by additionally creating new samples from the minority class [24, 25]. The traditional data mining algorithms suffer from computational deficiency, and sampling is an effective data reduction technique to reduce the computational cost and speed with high efficiency. Among the various random sampling methods, the stratified sampling technique suits the need of this work of dividing the available dataset into various strata and picking a random item from each group as items in a stratum will have common characteristics [26]. This sampling method is widely used in human research. Affinity propagation (AP) does not require mentioning the number of clusters to be formed as they were formed through message passing and so the exemplars computed from AP are the representatives of all other data points in the cluster. These exemplars are used to train the model [27].

Many research activities were carried out to classify emotions in the given dataset with different performance evaluators such as minimum error, precision, f-score, accuracy, and *p* value using many classifiers [28, 29]. In our research, the artificial neural network (ANN) is used for the classification of the EEG data as its results are more promising than the existing classification algorithms [30] and affinity propagation-based stratified sampling methodology along with the ANN suggested in this study was compared with the support vector machine (SVM) and random forest (RF) through various metrics.

The classical EEG signal classification as in various research papers involves the following steps: signal preprocessing->feature extraction->classification. But those algorithms failed to work efficiently as many other important concerns such as high-dimensional data, class imbalanced nature of the DEAP dataset, and the computation cost involved in training the classifier were not considered. Though many of these concerns were addressed separately in different research articles, there is no work on the hybrid model. In our proposed work, those limitations are addressed through suitable techniques and a multimethod hybrid model is proposed. The workflow of the proposed model works is as follows: signal preprocessing ->feature extraction ->feature selection ->class balancing -->stratified sampling->classification. The most highlighted innovative point of this research work is the usage of affinity propagation, a clustering algorithm as a stratified sampler. Representative samples selected through this method have more resemblance to the population than traditional sampling algorithms.

The key contributions of this research work are listed as follows:

- In our study, a set of signal processing steps were used in preprocessing the EEG signal preceding its analysis. We reached comparative performance results of the discrete wavelet transform (DWT) and independent component analysis (ICA) and concluded appreciating the better performance of the DWT in de-noising the EEG signal.
- As real-life signals are nonstationary, time-frequency analysis is used to localize them. The traditional methods are the usage of Fourier transform and Wavelet transform. Due to the limitation of the Fourier transform on resolution, the discrete wavelet transform (DWT) is chosen as apt for the EEG signals. The most significant 15 features (10 time domain +5 frequency domain) of wavelet coefficients from the five sub-bands which are the results of signal decomposition were extracted as suggested in many studies.
- As a feature selection technique, the Pearson correlation coefficient (PCC) method is applied in which the correlation between features is calculated to reduce the dimensionality of data and the top 10 features are selected.
- DEAP dataset suffers from the serious drawback of overfitting due to imbalanced classes and to overcome it, an adaptive synthetic sampling approach (ADASYN) is used that differs from SMOTE which fails to consider lower density areas when upsampling minority classes.
- To improve the performance and reduce the computation cost involved in training with very large data, stratified sampling is performed and representative elements were involved in training the classifier. Among the various sampling techniques, better coverage of the population is achieved by stratified sampling as the researchers can ensure that all of them are represented in the sampling. The general steps of the cluster-based sampling method involve some sampling scheme to decide on the number of representative samples and later applying the clustering algorithm, clusters are formed. The existing system increases the sampling complexity as the number of representative samples must be explicitly defined before clustering as that of the K-means and K-mediods and there is a requirement to fit the left-out sample objects. To overcome this, the affinity propagation (AP) is used which forms clusters by passing messages among the data points and the exemplars of the final iteration are taken as representative samples. This increases the efficiency of stratified sampling on large data and a comparison of the performance of AP with K-means and K-mediods is done in this study.
- Finally, experimental studies were conducted on three machine learning algorithms: SVM, ANN, and RF. Extensive experiments show that the fusion model of an AP-based sampler with the ANN model outperforms the state-of-the-art models.

## 2. Structure and Literature Review

The structure of this paper is such that Section 2 comprises other authors' contributions related to this research work and Section 3 includes the modular structure of the AP-ANN framework. In Section 4, the proposed model is implemented and its results are discussed, and Section 5 presents the detailed summary of the research work with limitations and future enhancements were suggested. Recognizing stress from the EEG signals is an interesting research topic for the past few years due to the increase in patients with depression, and there was a continuous urge to find a technological solution for it. There were many research works carried out trying to improve the output of the classified result. All the below-mentioned papers make use of the DEAP dataset for their research work proposing various feature extraction, feature selection, and emotion classification techniques.

Giuseppe Placidi et al. [31] proposed the classification of emotions using the DEAP dataset. From these participants, the relaxing phase EEG signals were obtained. The signals were decomposed using wavelet decomposition to approximation and detailed coefficients. The SVM classifier was used on the features extracted using the principal component analysis (PCA). Abeer Al-Nafjan et al. [32] proposed two emotional models of which the dimensional emotion model was used for emotion recognition which includes valence and arousal relation. The deep neural network and random forest classifiers were used to classify emotions, the feature extraction used time-frequency features and frontal asymmetry features, and results show that the DNN performs better than the random forest.

Jingxin Liu et al. [33] in their suggested model extracted the time, frequency, time-frequency, and wavelet domain-based features, and the mRMR algorithm was used for feature selection. The classification algorithms used were the random forest and KNN. Sukriye Kara and Ergin [34] used the DWT technique as a preprocessing algorithm, and the SVM was used as a classification algorithm. The features such as entropy, energy, and the standard deviation were computed. The different pairs of features were used for training. The energy feature with the SVM classification algorithm showed good accuracy in the detection of epilepsy. Sachin Borse [35] in their suggested model used the ICA and DWT for de-noising the EEG signals. The DWT decomposes the signal and applies thresholding to the decomposed signals. The ICA transferred the input signal into independent components and rejected the component with more noise. The whitening process was done before doing the ICA process to make the input signals uncorrelated.

Prashant Lahane and Thirugnanam [36] used Teager–Kaiser energy operator for the feature extraction, and classification tree, the K-nearest neighbor, and the neural network classifiers were implemented with the conclusion that the TKEO gives better accuracy than kernel density estimation and relative energy. Princy et al. [37] explained the statistical method for artifact removal from the EEG signals using the wavelet transform technique. The wavelet transform method analyzes the signals with low noise amplitudes so that they could be removed from the original signals by selecting the best wavelet to decompose the signal. The removal of artifacts from the EEG signal using the wavelet transforms was done by detecting its spikes without taking into the consideration of signal-to-noise ratio. Gaikwad [38] in their paper analyzed the effects of stress, and a methodology to detect the stress using the EEG signals was discussed. The phases involved in capturing the real-time signals from the NeuroSky Mind wave kit were explained, and the Fourier transform (FFT) was used as a preprocessing algorithm. The eSense meter, an analysis method, was used to convey if the user was in stress mode or without stress mode effectively. Bhuvaneswari and Satheesh Kumar [39] identified that the SVM machine kernel was used to classify the positive and negative values of arousal and valence.

Wolpaw et al. [40, 41] proposed the brain-computer interface methodology for providing communication capabilities for people who were suffering from neuromuscular disorders, and the differentiation of dependent BCI from independent BCI was made. Abin et al. [42] proposed a smart home environment adjustment system that was based on the EEG and IoT technology. The proposed system detected the cognitive state of the person (alert or drowsy), and based on it, it controls the devices in the environment. Ankita Tiwari [43] explained the usage of Lab VIEW for stress management using BCI. The NeuroSky Mind wave sensor was used in the system for the acquisition of the signal from the human brain, and their proposed system also includes an android application that helped to reduce stress by suggesting yoga and music after getting the SMS. Thejaswini et al. [44] reviewed two publicly available datasets (DEAP and SEED) that used the DWT for feature extraction and the SVM for classification and obtained the final output by channel fusion.

A detailed study on feature selection and feature extraction methods was performed for different datasets. Khan et al. in their paper [45] suggested a hybrid feature extraction method on the fusion of many known features such as GDC, RCC, and PseTNC and proposed an optimized DNN achieving 95.81% accuracy. The study in [46] explored the traditional feature selection methods and proposed the UFS-UDR method, and in [47, 48], classification of RNA sequence and efficient feature extraction from that data using the iEnhancer-DHF model which works on DNA samples were discussed. In paper [49], a two-stage gene selection method is proposed as the solution for the feature extraction problem and the SVM and RF were the classifiers used. Muhammad Ali et al. [50] analyzed the ANN and SVM classifiers on the stock dataset and proved that the ANN performs better. In [51], the RPOS feature selection method is proposed and its performance on the RF, SVM, and KNN is analyzed. Ishfaq Ali et al. [52] in their research used a data-driven approach to decide on the number of clusters, K in the K-means clustering algorithm, and in [53], the KNN-based ensemble method is proposed and performance is evaluated.

Samarth Tripathi et al. [54] proposed two classification models, deep neural network and convolution neural network, where the prepared data with 99 features were given as an input to the classifier DNN, and for the CNN, the DEAP dataset was converted into a 2D image, to make the CNN learn from the image for classification. It was modularized to prove the efficiency of neural in emotion classification. Ahmad and Olakunle [55] used the discrete wavelet packet transform (DWPT) in the work and the feature extracted in the work was entropy. It was concluded that compared to other statistical features like power and energy, entropy provides good accuracy. Pascal Ackermann et al. [56] extracted the features such as HHS, HOC, and STFT. The feature selection algorithm used was mRMR which was best suited for categorical output class labels. The classifiers used were the random forest and SVM. The output labels of classification were anger and surprise. The random forest was found to be the best compared to the SVM in that study.

## 3. Modular Structure of the AP-ANN Frameworks

The process involves developing a system to detect stress based on human emotions. In this proposed work, the pub is being used. The DEAP dataset contains 32 files, one per participant in a. dat or. mat format. Two arrays are generated for each participant as shown in Table 1. After gathering raw EEG data, preprocessing is performed on the data.

**Figure 1:**
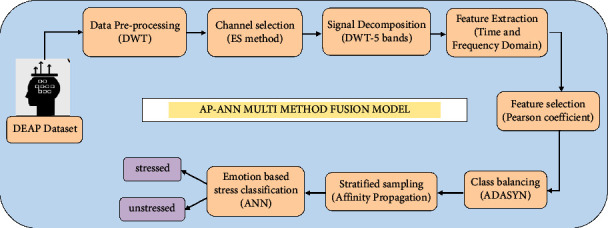
AP-ANN multimethod fusion model.

The dataset is raw such that it contains noise and artifacts; hence, it must be preprocessed to reduce the effect of this signal on feature extraction. Not all the channels contribute to emotion identification, so suitable channels are selected, and then, the multidomain feature set of time and frequency is obtained from the preprocessed signal. The most significant features that contribute to emotion identification are selected, and Russell's valence-arousal model of emotions is applied to the class label output. Before training, since the dataset is of high dimension and class is imbalanced, class balancing and sampling algorithms were applied to help in improving the dataset after which it is given as input to the classification models.  Figure 1 illustrates the proposed methodology.

DEAP is a database that is publicly available for the analysis of human emotions that contains the EEG and physiological signals from 32 participants that were collected while watching 40 one-minute videos, and the participants were asked to mark their real emotions on a five-level scale as valence, dominance, arousal, like, and familiarity.

### 3.1. Channel Selection and Signal Decomposition

Our goal of work is to recognize emotion from the EEG signals in the DEAP dataset. Using all the channels of the 10 × 20 system will result in data redundancy and an increase in computational time. So, in the proposed work, as Omid Bazgir et al. [57] suggested in their paper, only the frontal lobe channels are selected for research as it is proved that the left and right frontal regions of the brain contribute to emotion more than other channels. As a part of the experiment setup, the channels were selected as different pairs; an experimental selection (ES) of the channel is done, and the channels which respond the most during emotional change are detected and used in the research. The channels that were selected are FC1, FC2, FC5, FC6, F3, F4, F7, F8, FP1, and FP2. After the collection of the EEG data, it is preprocessed which is the process of removal of noise and artifacts from the raw brain signals without losing the original data or information. Preprocessing also includes the process of smoothing the brain signal. In the proposed work, the DWT and ICA performance is compared to the noise and artifact removal from the obtained brain signal.

#### 3.1.1. Discrete Wavelets Transform (DWT)

**Figure 2:**
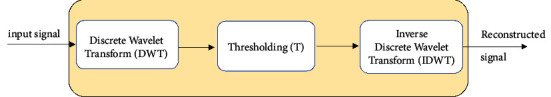
Stages in discrete wavelet transform.

In many scientific and engineering applications, discrete wavelet transform (DWT) is used as a signal processing tool. In wavelet transform, scaling functions and wavelet functions that are related to low-pass and high-pass filters, respectively, are involved. The DWT is used to decompose, de-noise, and recompose the EEG signal. The DWT algorithm considers the input signal as a wavelet as it involves both frequency and time domains so that the time at which the variation occurs at maximum and minimum in the signal can be found. It gives spectral information about both the frequency and time domain, whereas the other processing techniques such as fast Fourier transform are only for frequency domain analysis. The de-noising done by discrete wavelet transform is more efficient than other techniques as the de-noising of the signal is made without losing the original characteristics of the signal, because the de-nosing is performed after the decomposition of the signal. On the completion of the de-noising, the reconstruction of the signal is done to obtain the original noise-removed signal. The de-noising process involves the steps as shown in Figure 2.

**Figure 3:**
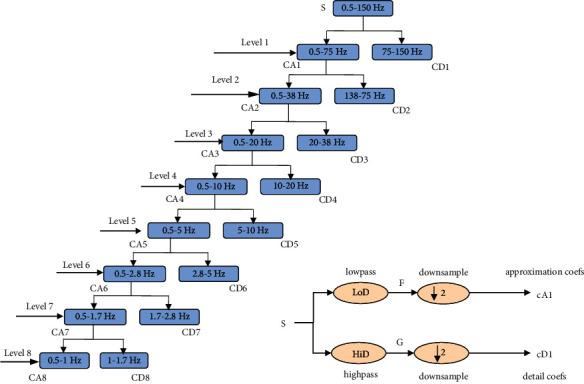
8-level decomposition of the EEG signal.

In the proposed work, the DWT algorithm has been used to split the EEG signal acquired from DEAP into approximation and detail coefficients using the filtering method. Usage of appropriate wavelet function and setting up the number of decomposition levels are the deciding factors of DWT performance. The wavelet family contains different types of wavelets such as Daubechies, Haar, Symlet, Mexican, Hat, and Morlet [58]. In the proposed work, the Daubechies-8 wavelet is chosen for wavelet analysis, and eight-level decomposition is preferred because it is considered to be more effective for signal de-noising compared to other wavelet families. As compared and analyzed by previous studies [59, 60], Daubechies is best suitable for analysis of the EEG signal due to its smoothening feature [61] and its accuracy is compared with other mother wavelet families. The filters used in the DWT algorithm are the low-pass filter and high-pass filter as shown in Figure 3. After acquiring the low pass filter's approximation coefficient and the high-pass filter's detail coefficient at level 1, the level 2 coefficients can be obtained by applying the same decomposition procedure to the level 1 approximation coefficient. Similarly, the outputs from low-pass filters at each level are decomposed further. Thus, the acquired EEG signal from DEAP is decomposed into eight levels of coefficients that are CD1, CD2, CD3, CD4, CD5, CD6, CD7, and CD8, and an approximation coefficient is CA8 as in Figure 3. The thresholding technique is applied to the obtained detail and an approximation coefficient. The threshold value is calculated for each of the coefficients using the formula as follows:

(1) $$\begin{array}{c} \text{threshold}=\text{sqrt}\left(2*\log\left(n\right)\right). \end{array}$$

Soft thresholding is applied [61] after calculating the threshold value in which coefficients having values higher than the threshold value are minimized towards zero. Thus, the de-nosing of the coefficients is done, and the de-noised detail and approximation coefficients are obtained.

After thresholding, the reconstruction is done on the de-noised coefficients to obtain a de-noised EEG signal. The preprocessed EEG signal is partitioned into five frequency sub-bands, alpha, beta, gamma, theta, and delta, as shown in Table 2.

#### 3.1.2. Independent Component Analysis (ICA)

To compare with DWT, the EEG data are de-noised by ICA using the EEGLAB toolbox. The ICA tries to maximize independence by linearly transforming the input signal into subcomponents such that the mutual information between these subcomponents is zero. This method assumes that each of the subcomponents generated is independent of each other. The important aspect of ICA is that the number of input signals (S) of ICA and the number of subcomponents (C) generated must be the same. The other two cases are shown as follows:

(2) $$\begin{array}{c} \text{source}<\text{components}–\text{overdetermined}. \end{array}$$

(3) $$\begin{array}{c} \text{source}>\text{components}–\text{underdetermined}. \end{array}$$

The EEGLAB toolbox is used for implementing ICA-based de-noising. A participant dataset acquired from DEAP is loaded into EEGLAB. The ‘runica' algorithm is selected in the EEGLAB to decompose the input signals into subcomponents. The input signals are acquired from the 10 selected channels of the DEAP dataset; therefore, 10 independent subcomponents are generated. The order of the subcomponents generated is based on the variance of each component compared to that subcomponent with higher variance is rejected by the “pop_subcomp” function in EEGLAB. After the components are removed, the subcomponents are reconstructed to obtain the original 10 channels signals which are free from artifacts and noise.

### 3.2. Feature Extraction

It is a process of transforming the original raw data into an optimal set of features for processing. In the proposed work, after preprocessing the EEG signal, the features are extracted from the de-noised EEG signal for the further classification process. The feature set contains time domain and frequency domain-based features as shown in Table 3, extracted from the EEG signals. Time domain-based analysis is a statistical analysis that gives more information about the signal amplitude variation. Frequency-domain analysis gives more information about patterns in the signal. As many research articles suggest various features that perform well for the EEG signals, for time domain-based analysis, the statistical features as suggested in [34] were extracted, and frequency-domain features such as energy, log energy entropy, Shannon entropy, power spectral density, and absolute power as suggested in reference [47] were extracted and used in research.

These features along with median and mode as statistical features contribute to 15 features. As there are 5 frequency bands, the features were calculated for each band, thus preparing the multidomain feature set. Though frequency- and time-domain features have their limitations and advantages, the proposed multidomain feature set increases the accuracy of the classification. Thus, the feature set initially contains 75 features in total, of which 50 are time-domain features and 25 are frequency-domain features.

### 3.3. Feature Selection

The data with irrelevant or trivial features may lead to a reduction in the efficiency and performance of the model. Hence, there is a need for selecting significant features that have more impact on the prediction accuracy. Feature selection is the process of selecting the salient features from the given dataset. It is used to eliminate the irrelevant features in data, thus improving the performance of learning and reducing the time consumed to train the model. In the proposed work, filter feature selection is used because it works fine with large datasets containing many features while the wrapper technique is expensive to run and complex for large datasets. In the proposed work, the Pearson correlation coefficient (PCC), a filter-based feature selection, is used. Correlation can be used to identify how one or multiple features are associated with other features.

As the first step of feature selection, a temporary feature set with a total of 15 features is prepared by selecting the features of the preprocessed signal; then, the correlation is calculated between each feature to the output label using the Pearson correlation coefficient formula C as follows:

(4) $$\begin{array}{c} \;C=\frac{\sum_{i=1}^{n}\left(x_{i}-\bar{\text{x}}\right)\left(y_{i}-\bar{\text{y}}\right)}{\sqrt{\sum_{i=1}^{n}{\left(x_{i}-\bar{\text{x}}\right)}^{2}}\sqrt{\sum_{i=1}^{n}{\left(y_{i}-\bar{\text{y}}\right)}^{2}}}, \end{array}$$

where *n* is the number of samples, *x*_*i*_ and *y*_*i*_ are the ith data values of two sets {*x*1, *x*2,. *xn*} and {y1, y2,.yn}, and $\bar{x}$ and $\bar{y}$ are the mean values. The correlation value (C) lies between -1 and +1, and if the score is near to +1, indicates that there is a strong positive correlation between features; that is, if one feature increases, another feature also increases or if one feature decreases, other feature also decreases. A correlation score near to -1 indicates a strong negative correlation; that is, if one feature increases another feature decreases and vice versa. The correlation score of 0 indicates there is no relationship. In the proposed work, the correlation values of the 15 features are obtained from the Pearson correlation coefficient. Then, these values are sorted in decreasing order, their ranking indexes are found, and the top 10 features are listed in Table 4.

Initially, the first feature is selected, and the feature set is expanded by adding the next feature in order. This process is called forward selection. Each time a feature is added, it is evaluated, and the prediction accuracy is calculated. The process is continued until there is no improvement in the prediction accuracy and the best feature set is obtained. In the proposed work, with a subset of 10 features, the feature selection process is stopped.

### 3.4. Russell's Valence-Arousal Model

**Figure 4:**
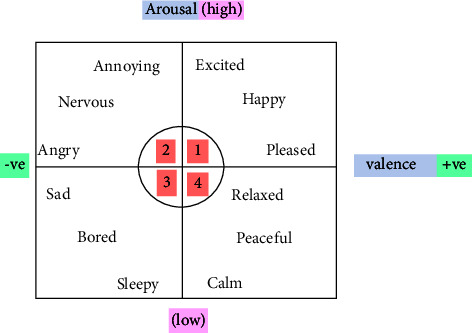
Russell's valence-arousal model (1: happy, 2: angry, 3: sad, and 4: relaxed).

In the proposed work, emotions have been used to classify stressed and unstressed states among people. The people with positive emotions are in an unstressed state, while people with negative emotions are in a stressed state. To determine the output labels for the feature set, Russell's valence-arousal model of emotions is used as shown in Figure 4.

**Figure 5:**
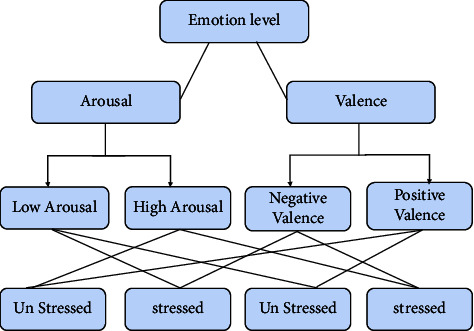
Stress calculation.

In the DEAP dataset, the responses from participants were labeled as various emotions in the valence-arousal model, each taking a value of *x* where *x* takes a value from 1 to 9. A threshold value of 5 is assigned so that the labels have been classified as high and low. In the proposed work, if the valence is -ve and arousal is high, or the valence is -ve and arousal is low, then the output label is determined to be “1” and concluded as a stressed state. If the valence is +ve and arousal is high, or the valence is +ve and arousal is low, then the output label is determined to be “0” and concluded as an unstressed state as shown in Figure 5.

Thus, the final dataset contains a collection of the feature set with selected features and its corresponding output label with “1” (stressed) or “0” (unstressed). The final prepared dataset is used to classify a person's stress-based valence and arousal values.

### 3.5. Class Imbalance Reduction

The DEAP dataset normally consists of 40 videos and 32 participants. Hence, there are 1280 sample signals for each channel. The labels of these samples are significantly imbalanced. The proposed work is a two-class classification where the two classes are 0 (stressed) and 1 (unstressed) which are not in a uniform distribution. So, the upsampling of the minority class 1 is done to make the dataset a balanced one. The algorithm ADASYN (adaptive synthetic approach) is used for resampling the dataset by creating new synthetic samples. The linear interpolation method is being used to scale up the minority class examples by creating new examples, and this algorithm is an oversampling technique for the minority class which is mostly used for imbalance problems.

### 3.6. Stratified Sampling

As the proposed model is dealing with the data of high dimensionality, using all the records to train the model increases the computational time and thus an efficient sampling method is suggested to find the optimal set of records to train the model. Stratified sampling is a sample selection technique in which the records of interest are being subdivided into homogeneous clusters or strata and a representative from each cluster is taken as a sample for analysis [62]. In this project, affinity propagation is being used for the formation of strata, which is a clustering algorithm that does not require specifying the number of clusters prior as it is based on message passing between the exemplars. An exemplar is the unique data point that forms the centre of the cluster. This similarity is taken as input, and it is calculated using the negative Euclidian distance square between each data point as follows:

(5) $$\begin{array}{c} s\left(a,\;b\right)\;=\;-\;x_{a}-x_{b}{}^{2}. \end{array}$$

The similarity *s* (*a, b*) indicates how well point *b* is suited to act as an exemplar for point a. The diagonal of *s* (*a, b*) where *a* = *b* is known as “preference” which has control over the number of clusters generated. Once the similarity between data points is found, the messages which include responsibility and availability values are exchanged between the data points. The responsibility *r* (*a, b*) is represented as follows:

(6) $$\begin{array}{c} \text{r}\left(\text{a},\text{b}\right)\;=\text{ s}\left(\text{a},\text{b}\right)\;–\;\begin{array}{c} \text{max}\\ b\neq b \end{array}\left\{\text{avail}\left(a,b\right)+s\left(a,b\right)\right\}, \end{array}$$

where *b* is competing for exemplar.

The responsibility *r* (*a, b*) represents the messages sent from the data point a to the exemplar *b* indicating how well the point *b* is to be an exemplar for point a. The availability avail (*a, b*) represents the messages sent by exemplar *b* to point a indicating how well a selects *b* to be its exemplar. The availability avail (*a, b*) is represented as follows:

(7) $$\begin{array}{c} \text{avail}\left(a,b\right)\;=\text{ min }\left(0\right.,r\left(b,b\right)\;+\underset{a∄\left\{a,b\right\}}{\sum }\text{max}\left(0,r\left(a,b\right)\right), \end{array}$$

where a ≠*b*. The important parameter in affinity propagation is the damping factor *λ* which avoids the numerical oscillation while exchanging messages. The addition of the damping factor to the responsibility and availability are shownas follows:

(8) $$\begin{array}{c} \text{res }=\;\left(1-\lambda \right)\text{ res }+\lambda \text{ res}, \end{array}$$

(9) $$\begin{array}{c} \text{avail }=\;\left(1-\lambda \right)\text{avail }+\lambda \text{ avail}. \end{array}$$

The damping factor can have a value from 0.5 to 0.9, and in the proposed work, the damping factor is fixed to 0.5. The responsibility and availability matrix are updated until the maximum iterations are reached, or values fall under a certain threshold, or values remain constant. Once the updation of responsibility and availability matrix is completed, the final exemplars are computed by calculating criterion matrix which is represented as follows:

(10) $$\begin{array}{c} c\left(a,b\right)\;=\text{ res}\left(a,b\right)\;+\text{ avail}\left(a,b\right). \end{array}$$

Here, *b* with the highest criterion value in each row of *c* (*a,b*) is an exemplar for data point a. The data points that have common exemplars are grouped under the same cluster. In the proposed work, the training dataset has been prepared using the AP to train the classifier model to increase its efficiency and performance. The population is divided into strata through affinity propagation and thereby exemplar which is a particular data record that represents the entire data records chosen from each stratum from which better accuracy and performance can be obtained. Similarly, the output label of the exemplar is obtained by selecting labels of data records that appear most in the attribute.

### 3.7. Emotion Classification for Stress Detection

In the proposed work, a pattern recognition network which is a feed-forward backpropagation neural network (FFBPNN) is trained to classify the inputs depending on the output. The flow of information starts from the input node and then to the hidden layer and finally to the output nodes in the feed-forward network. The backpropagation algorithm is a training method of the neural networks that compares the actual outputs with the expected outputs and the error is calculated, and based on it, the weights of layers are adjusted backwards from the output layer to the input layer.

In the proposed work, the neural pattern recognition toolbox of the MATLAB framework has been used to train the feature set. As an initial step, the pattern recognition network randomly assigns weight and biases to the nodes in the neural network. As the extracted samples with their features selected are given as input to the network input layer, then the input vector is divided independently at a ratio of 4 : 1 (80% training set and 20% test set). This ensures that unknown samples were fed into the classifier during testing, and thus, the performance of the model is analyzed. The ANN model is compared with the random forest and SVM and proved to provide better classification results; thus, the neural network classification model along with affinity propagation is used to detect the stress of the participants when watching the videos (See Algorithm 1).

## 4. Results and Discussion

**Figure 6:**
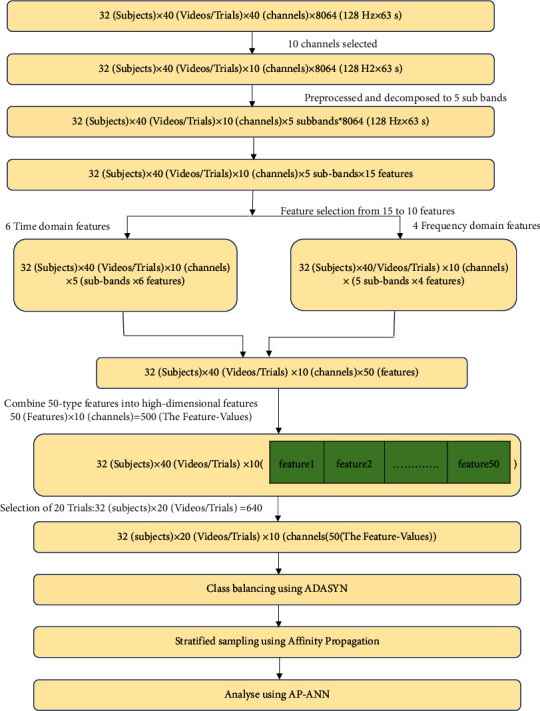
Dataset processing workflow diagram.

In the process of finding the stress of the participants from the DEAP dataset, initially, the analysis of the dataset is done, the workflow is framed, and the workflow diagram of the AP-ANN model for the DEAP dataset emotion classification process model is as given in Figure 6.

**Figure 7:**
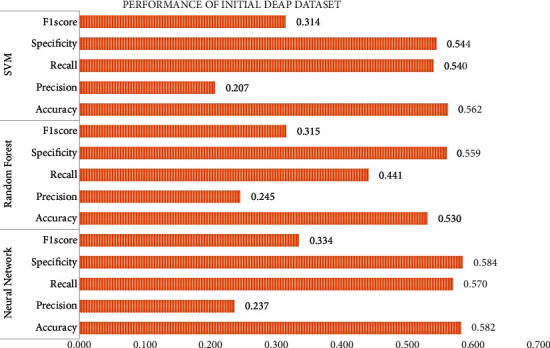
Initial DEAP dataset classification performance.

Initially, a dataset is prepared using the raw EEG recordings of 40 channels of 32 participants in the DEAP database where each of them must take up 40 trials and 15 features for 5 sub-bands, which yield to a total of 75 features obtained by DWT from the EEG recordings of DEAP. With the initial feature set, a matrix of 51200^*∗*^75 is formed which is given as input to the three classifiers (neural network, random forest, and SVM) and Figure 7 shows the performance comparison.

**Figure 8:**
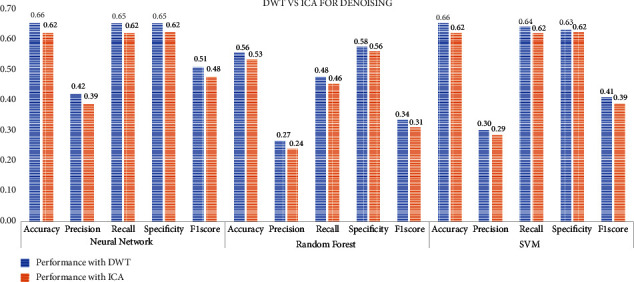
De-noising using DWT vs ICA.

Then, a preprocessing step is involved after selecting the most important 10 channels that contribute to the identification of stress levels. This step involves de-noising the signal, a comparison is done between the two methods DWT and ICA, and again the performance is compared on the various performance metrics as shown in Figure 8.

**Figure 9:**
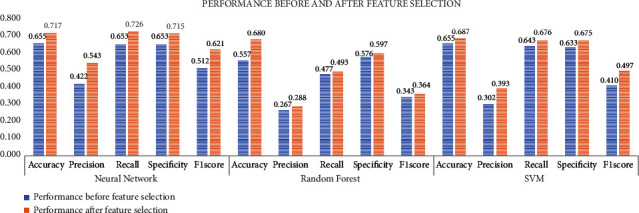
Performance before and after feature selection.

Still, the classification accuracy of this preprocessed dataset is not appreciable as there was a class imbalance, and the dataset is high dimensional. Dimensionality reduction is done, by selecting the 10 significant features using the Pearson coefficient method. After the completion of the selection process, 50 features are calculated with selected 10 channels for 32 participants. The feature vector is given as an input to the classifiers (SVM, random forest, and neural networks), and a detailed comparison before and after the feature selection is done and is shown in Figure 9.

Now, the dataset is (32 participant ^*∗*^ 40 trial ^*∗*^ 10 channel) ^*∗*^50 features, i.e., 12800^*∗*^50. The performance of the classifier is checked for 10, 20, 30, and 40 trials, and it was found that no huge variation in the performance standards happens, as the trial increases from 20 to 40; i.e., the dataset is (32 participants ^*∗*^ 20 trial ^*∗*^ 10 channel) ^*∗*^50 features, i.e., 6400^*∗*^50, and so first 20 trials were selected for further process. This comparison study is given in Table 5. Moreover, this process reduces a lot of the training time, but still, the precision of the algorithm is very low due to the imbalanced nature of the classes.

**Figure 10:**
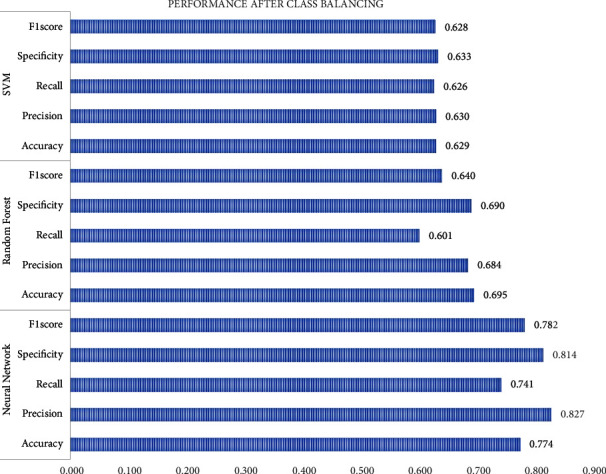
Performance after class balancing.

In the process, overfitting seems to be one stopping factor for performance enhancement, and the dataset is balanced with the ADASYN for preventing the classifiers from overfitting and to improve the classification rate. After class balancing, the precision is greatly improved as in Figure 10.

**Figure 11:**
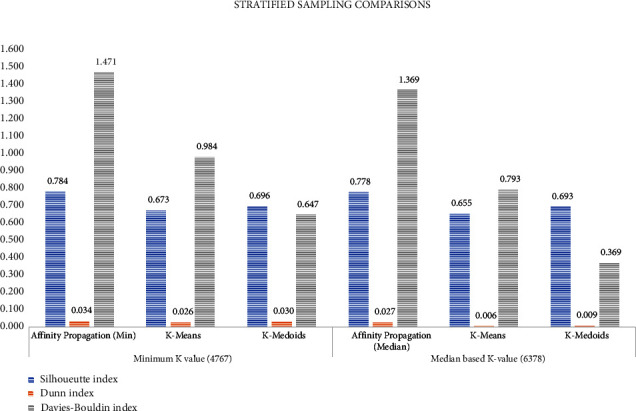
Performance analysis of stratified sampling algorithms.

After the ADASYN process, the addition of 4095 samples of the stressed class is added synthetically resulting in our final dataset of 10495^*∗*^50 features and one output label. The computational time taken for the entire process seems to be huge when given to the classifier and so a stratified sampling approach is used to handle it. Various cluster-based sampling methods like the AP, K-mean, and K-medoid were experimented with stratified sampling method, and their clusters are validated with the Davies–Bouldin index, Dunn index, and Silhouette index. Comparison is shown in Figure 11. Based on the performance parameter, the affinity propagation clustering can be either based on minimum preference value or median preference value. The performance of both the preference value is analyzed in the same figure.

**Figure 12:**
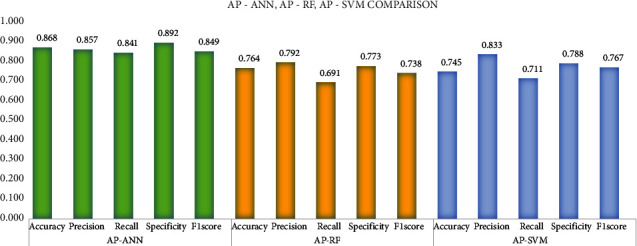
Performance analysis of AP-ANN, AP-RF, and AP-SVM.

Affinity propagation (AP) shows better clustering consistency as a stratified sampler and its preference along with various classifiers such as the ANN, RF, and SVM are illustrated in Figure 12. The performance is analyzed on the five evaluation metrics among which accuracy and specificity seem to be far better in the AP-ANN compared to AP-RF and AP-SVM.

**Figure 13:**
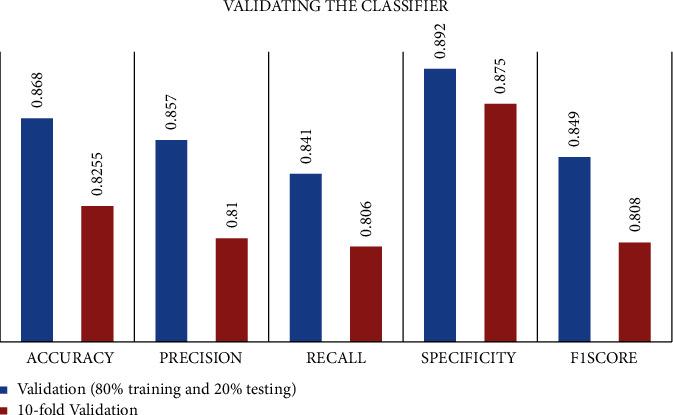
10-fold validation of the classifier.

The 10-fold cross-validation [63] which is the error estimation method generally has a lower bias than other methods and is not appropriate to classify the original unbalanced DEAP dataset, but our ADASYN balanced DEAP dataset can be validated, and the results of this 10-fold validation are compared and illustrated in Figure 13.

The affinity propagation (AP) is the best-stratified sampler, and having its preference value as minimum (min) along with the neural network classifier gives better performance. As our proposed system yields better accuracy, high precision, better recall, and specificity, and a decent F1 score, a performance comparison of the proposed AP-ANN model with the performance results of various algorithms that used the DEAP dataset in various other research papers is carried out and shown in Table 6.

## 5. Conclusions

The proposed multimethod fusion model based on the AP-ANN approach for stress detection by analyzing the bad and unhappy emotions provides more promising results, and the findings are DWT provides better performance compared to ICA in de-noising. It is used to extract features of both time and frequency domains, and highly correlated features that were selected increase the system's efficiency. The upsampling of minority classes using ADASYN removed the threat of overfitting, and performing stratified sampling using the AP clustering ensures best fit representatives are used to train the classifier model and attain greater performances. The classification accuracy has been compared among the most significant three classification algorithms such as the SVM, neural network, and random forest among which the neural network has achieved a high accuracy of 86.8% which is 9% better than the result obtained without affinity propagation, 16% better than the result obtained without ADASYN and AP, and 29% better result than that of the classification of the preprocessed data. Furthermore, the proposed method has some limitations as, in the DEAP dataset, a single evaluation may not be enough to rightly represent the emotional state of the participants as the video extracts are played for 60 sec, there is a tremendous chance for many emotional states to be evolved in that period. When dealing with imbalanced classes using the ADASYN algorithm, adjusting and refining the data have its boundaries. Though balanced data classes have an experimental need, creating new data can never replace the original features. Stratified sampling generally cannot be used in all studies as it has a drawback that each member of the population must be studied individually to find the best representative sample and also finding an exhaustive list of representative samples is very challenging. The future scope of research in this domain is the fusion of other physiological data from various sources that can be used along with the EEG signals as a hybrid model to improve the performance of emotion classification.

## Figures and Tables

**Algorithm 1:**
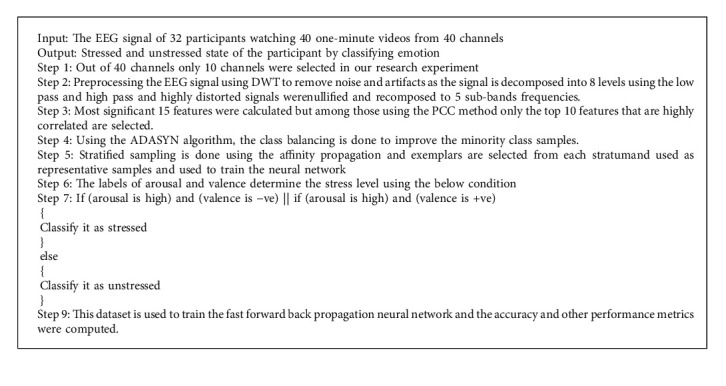
The AP-ANN model for classifying stress through human emotion.

**Table 1:** DEAP dataset description.

Array details	Shapes	Contents
EEG data	40 × 40 × 8064	Trials(videos) *X* channels *X* data readings
Labels in dataset	4 × 40	Labels *X* videos

**Table 2:** Decomposition of sub-bands.

Decomposition levels	Sub-band signals	Frequency bands (Hz)
5	CD5 (gamma)	30–60
6	CD6 (alpha)	15–30
7	CD7 (beta)	8–15
8	CD8 (theta)	4–8
8	CA8 (delta)	0–4

**Table 3:** Features formula and description.

Features	Formulas	Descriptions
Variance	$V=1/N-1\overset{N}{\underset{i=1}{\sum }}{\left|A_{i}-\mu \right|}^{2}$ where *μ*-mean of A, $\mu =1/N\overset{N}{\underset{i=1}{\sum }}A_{i}$	Variance is used to show the distribution of the EEG data points of the signal from their actual mean value.
Standard deviation	*S* = $\sqrt{1/N-1\overset{N}{\underset{i=1}{\sum }}{\left|A_{i}-\mu \right|}^{2}}$*μ*-mean of a vector, $\mu =1/N\overset{N}{\underset{i=1}{\sum }}A_{i}$	The standard deviation describes the fluctuation of the EEG signal from the mean value. The standard deviation if the value is higher indicates that the EEG signal data points are very close to the mean.
Minimum	Min (A)	The minimum of the EEG signal over the segment is calculated.
Maximum	Max (A)	The maximum of the EEG signal over the segment is calculated
Mean	$\mu =1/N\overset{N}{\underset{i=1}{\sum }}A_{i}$	The mean of the EEG signal over the segment is calculated.
Root mean square	$\sqrt{1/N\overset{N}{\underset{i=1}{\sum }}{\left|A_{i}\right|}^{2}}$	The square root of the arithmetic mean of the square of the EEG signal is calculated.
Skewness	*S* = *E*(*x* − *μ*)^3^/*σ*^3^	The skewness is the measure of distortion of the EEG signal data from the symmetrical distribution. The symmetrically distributed data will have skewness 0.
Kurtosis	*K* = *E*(*x* − *μ*)^4^/*σ*^4^	Kurtosis measures the complexity of the EEG data points. The higher kurtosis indicates the sharp peak of the signal is at the mean point.
Energy	E(x)=(sum(x^ 2))	The wavelet energy describes the percentage of the energy of different frequency bands of the EEG signal.
Log energy entropy	E(s) = $\underset{i}{\sum }\log\left(s_{i}^{2}\right)$	Log energy entropy is to find the distribution of the energy of EEG signal
Shannon entropy	E(s) = $\underset{i}{\sum }s_{i}^{2}\log\left(s_{i}^{2}\right)$	The Shannon entropy is used to indicate the variation of the signal at each frequency scale.
Power spectral density	PSD = (1/*N*) ^*∗*^abs (×)^ 2	The PSD is used to identify brain wave differences in terms of frequency.
Absolute power	Power(×)= (sum(x ^ 2))/length(×)	The absolute power describes the power of the entire signal.

**Table 4:** Ranking of the features.

Ranks	Features
1	Standard deviation
2	Maximum
3	Shannon entropy
4	Power spectral density
5	Absolute power
6	Energy minimum
7	Variance
8	Skewness
9	Minimum
10	Kurtosis

**Table 5:** Performance comparison in selecting the number of trials.

No. of trials		10	20	30	40
Neural network	Accuracy	0.827	0.763	0.725	0.717
	Precision	0.323	0.476	0.498	0.543
	Recall	0.788	0.715	0.685	0.676
	Specificity	0.868	0.765	0.682	0.715
	F1 score	0.458	0.572	0.577	0.602

Random forest	Accuracy	0.827	0.739	0.707	0.680
	Precision	0.253	0.294	0.319	0.348
	Recall	0.638	0.515	0.455	0.493
	Specificity	0.601	0.541	0.539	0.527
	F1 score	0.362	0.374	0.375	0.408

SVM	Accuracy	0.806	0.784	0.766	0.757
	Precision	0.179	0.180	0.240	0.393
	Recall	0.256	0.343	0.445	0.676
	Specificity	0.736	0.695	0.680	0.665
	F1 score	0.211	0.236	0.312	0.497

**Table 6:** Comparison with other research works.

#References	Feature extraction-classification algorithms	Accuracy (%)
(Shon et al.)	GA- (KNN)	71.76
(Hasan et al.)	Boruta-(KNN)	73.38
(Arsalan et al.)	Wrapper FS- (MLP, SVM)	67.85
(Martínez et al.)	2-D AlexNet-(CNN)	84.60
(Asghar et al.)	DWT-BODF (SVM, KNN)	77.40
Proposed AP-ANN model	DWT-AP-ANN	86.80

## Data Availability

The data that support the findings of this study are available from the DEAP dataset, a dataset for emotion analysis using the EEG, physiological, and video signals in the following link: http://www.eecs.qmul.ac.uk/mmv/datasets/deap/download.html. The license of the dataset is for academic research only and not publicly available.
